# “We are at war”: The military rhetoric of COVID-19 in cross-cultural perspective of discourses

**DOI:** 10.3389/frai.2023.978096

**Published:** 2023-03-09

**Authors:** Paola Giorgis, Olena Semenets, Bilyana Todorova

**Affiliations:** ^1^Independent Scholar, IOW Dictionary, Turin, Italy; ^2^Department of Slavic Philology and Journalism, V. I. Vernadsky Taurida National University, Kyiv, Ukraine; ^3^Department of Bulgarian Language, South-West University “Neofit Rilski”, Blagoevgrad, Bulgaria

**Keywords:** COVID-19 and military rhetoric, online dictionary in other words, communicative strategies, political agendas, cross-cultural analysis, the media

## Abstract

At the outburst of the COVID-19 pandemic and all throughout its continuation in 2020 and 2021, the metaphor of ‘war' has been one of the most pervasive and recurrent globally. As an international, cross-cultural group of scholars and practitioners, we will analyze critically the communicative strategies enacted and the political agenda that they have meant to serve in Italy, Bulgaria, and Ukraine discussing both the cultural differences and the cross-cultural similarities of such a discourse that has been shaping the perception of our factual reality during the pandemic. Expressions like ‘We are at war', ‘Our heroes are fighting at the forefront', ‘We will win this war' and the like contributed to create symbolical cross-cultural responses that, by playing on emotions such as fear, uncertainty and, in some cases, national pride, contributed to the creation of a new state of reality, that of the “new normality”, calling for specific actions and behaviors. However, the war metaphor assumed different hues according to the country in which it was disseminated, up to the actual appointment of generals as governmental spoke-persons or organizers of the vaccine logistics, often combined with the construction and the mediatization of the archetypical hero fighting against the virus/enemy. To analyze how, all throughout 2020 and 2021, the military rhetoric was implemented and disseminated as the dominant discourse, we draw on Media Representations of the Real, on Rhetoric Studies on Manipulation, on Political Discourse, on Critical Discourse Studies, and on Susan Sontag's fundamental essay Illness as Metaphor. We discuss such rhetorical strategies as they originated from a discussion within our collective project in other words, an online dictionary that, besides critically analyzing contextualized keywords that (re)produce different forms of Otherness, offers creative proposals to reverse such narratives, and can be used as a free resource in different social and educational contexts (www.iowdictionary.org).

## 1. Introduction

This contribution discusses how the word “war” and the war-like metaphors were mobilized in public and political discourses to define the COVID-19 pandemic in Italy, Bulgaria, and Ukraine, also analyzing critically and from a cross-cultural perspective the different agendas they were meant to serve.

The Cambridge Dictionary defines the word “war” as follows: (1) armed fighting between two or more countries or groups, or a particular example of this; (2) any situation in which there is strong competition between opposing sides or a great fight against something harmful.^1^

After Putin's invasion of Ukraine, the first (literal) meaning of the word became active for Europeans again. However, the war rhetoric has continued to be used even at times of peace, and, in different situations, the metaphoric meaning of the word “war” has been activated. Metaphorical uses are so popular because they are a result of people's capacity to see the similarities between different domains and express them linguistically. They are very frequent in language as they occur “between 3 and 18 times per 100 words” (Semino, 2021, p. 50). Metaphors are both means of linguistic economy and more importantly, a means of human creativity. At the same time, they shape our thinking as “using different metaphors can lead people to reason differently about notions like time, emotion, or electricity” (Thibodeau and Boroditsky, 2011).

The influence of metaphorical use and its capacity to “shape the goals we seek, the plans we make, the way we act, and what counts as a good or bad outcome of our actions” (Lakoff, 2004, p. XV) is used broadly by politicians, PR specialists, journalists, and other professionals who rely on language not only as a means of communication but also as an opportunity to influence public opinion, as it empowers them to achieve their goals.

Indeed, metaphors help us make sense of complex events and have the capability to shape, orientate, and modify our actions and behaviors (Lakoff and Johnson, 1980/2003). The use of military vocabulary is a rhetorical device that relies on specific metaphors to convey determinate messages and meanings. In the case of COVID-19, the rhetorical use of the war metaphor functions at several communicative levels and serves several purposes. According to Thibodeau and Boroditsky's (2011) research findings, “metaphors exert their influence, by instantiating frame-consistent knowledge structures, and inviting structurally-consistent inferences,” and therefore the frame “Disease is a War” may influence people's perception of the pandemic. Narrating the pandemic as a state of war would, for example, make people accept more easily censorship, the military presence in the streets, the restriction of individual liberties, the silencing of dissent, and the enactment of social control. Some analysts, though, are cautious to take a resolute stance against the use of the war metaphor, since it could also inspire a sort of positive effect (e.g., fear can motivate people to pay more attention; Piazza, 2020, p. 91), prepare the public for hard times or inspire a renovated sense of unity and solidarity (Castro-Seixas, 2020)—yet: why promoting solidarity through a word that, by definition, is divisive? Words such as “care,” “community,” “aid,” would move representations and energies toward something *for* and not *against*, pointing to mutual collaboration between individuals rather than to the confrontation against an invisible enemy.

Yet, there is another point that has to be made. If we reverse the metaphor, other meanings appear. If a pandemic is a war, that means that also war is a pandemic. Implying that disease is like war, it also suggests that war is like disease, that is something which is not chosen but that rather happens as one of the natural incidents of the human condition. This is where the military rhetoric makes another point: culturalize disease and naturalize war.^2^ Such a naturalization also allowed us to assign “to the virus (COVID-19) the problems or crisis that were not generated by it” (Dias and Deluchey, 2020, p. 7) such as unemployement, the working poor, the cuts to social and health systems, and social inequalities, “transferring to the pandemic (a “natural phenomenon”) the responsibilities for the problems created by neoliberal, necropolitical governmentalities” (*ib*., p. 8).

But the constant appeal to military rhetoric also showed that another naturalization was in act—though, for once, with positive outcomes. Many studies (e.g., Williams, 2020; Esanu, 2021; Waylen, 2021) have highlighted the hypermasculinity/toxic masculinity that was exhibited in the war-like rhetoric connected to COVID-19, and how precisely such an attitude failed to address efficaciously the pandemic. The enactment of health measures was associated with “stereotypically feminine characteristics like weakness and vulnerability” (Esanu, 2021), while not wearing a mask, being proud of not respecting interpersonal distance, performing a profusion of handshakes, delaying the lockdown, ridiculing mitigating measures, became the visual and symbolic representations of a macho attitude that dare to challenge the enemy with bare hands. Among others, champions of this attitude were Trump, Johnson, and Bolsonaro—who, incidentally, all got infected. Conversely, the most effective leadership styles in managing the COVID-19 pandemic were those based on empathy, a community-focused approach, resilience, adaptability, the ability to collaborate as those enacted by several women leaders as New Zealand's Prime Minister Jacinda Ardern, Taiwanese President Tsai Ing-Wen and Norwegian Prime Minister Erna Solberg. “For once, women leaders have the advantage of gender expectations that are more suited to dealing with crises such as pandemics” (Williams, 2020, p. 24). Such evidence further indicates the urgent necessity of a more general change in the global styles of leadership, one that contrasts traditional, patriarchal and authoritarian leadership style, and moves toward “one that prioritizes communication, empathy, decisiveness and community” (*ib*., p. 25). After analyzing the different types of hypermasculinity displayed by Johnson, Trump, Putin, and Bolsonaro, from a feminist institutionalist (FI) approach Waylen (2021) rather speaks of “hypermasculine leadership traits” not confining them to man-only: female leaders can adopt hypermasculine leadership styles, while male leaders can opt for more caring and community-oriented leadership styles.

Another fundamental aspect of the war rhetoric, no matter if the word is used metaphorically or not, is its connection with power since it directly points to a “state of exception” where fundamental rights can be repealed and control can be exercised. It is indeed in and through language that unequal relations of power are constructed and reproduced, and discriminatory practices are exerted. Studies in Critical Discourse Analysis (CDA) have uncovered the power dynamics connected to language, analyzing how power is enacted, reproduced and resisted, through text or speech (Van Dijk, 2001, p. 43).

Therefore, referring to Lakoff and Johnson's CMT and the CDA approach we decided to investigate the role and the function of war metaphors in the conceptualization of COVID-19 pandemic in the countries where we live.

There is plenty of research concerning war metaphors during the COVID-19 pandemic (Castro-Seixas, 2020; Panzeri et al., 2021; Semino, 2021; Todorova, 2021a; Benzi and Novarese, 2022, etc). This contribution originates from discussions within our collective project *In Other Words*—*A Contextualized Dictionary to Problematize Otherness*, an online dictionary that can be used as a free resource in different social and educational contexts (www.iowdictionary.org). The dictionary critically analyzes contextualized keywords which have been shaping different forms of Otherness, juxtaposing some creative proposals to problematize and reverse such narratives. The dictionary pursues an integrated interrelation between theoretical reflections, societal issues, and the application of research in different real-life contexts. The dictionary has also a special section dedicated to the language of COVID-19 to show how, in different contexts and different countries, it has contributed to creating or reinforcing different forms of Otherness.

The following argumentation will analyze critically and cross-culturally the communicative strategies enacted and the political agenda that they have meant to serve in Italy, Bulgaria, and Ukraine, and how they have contributed to shaping the perception of our factual reality during the pandemic. Apart from the differences between the political, social, and healthcare situations of different countries, we also investigate the similarities in the speeches of public figures and in the actions of the governments. Political leaders of many counties used war rhetoric when talking about COVID-19 (Dada et al., 2021), i.e., it is a widespread phenomenon that needs more investigation and it has to be analyzed critically and comparatively to show both the common features and the peculiar ones. Moreover, as Semino (2021) states: “the establishment of martial law and or warlike powers for the executive in different countries reveals the potentially fuzzy boundary between the literal and metaphorical status of military references during the pandemic.” That is the reason why we will discuss the spread and the prominence of the military language used both metaphorically and how it will be shown—in some cases literally—in three different contexts.

## 2. Methodology/theoretical background

As it was mentioned, according to us the CMA and the CDA are the most appropriate perspectives for achieving the goals we have set. We are interested in war-like rhetoric uses connected to COVID-19 in public speech in our countries and military language is a means of expressing brute force and immense power. What is more important, power is crucial when we are talking about war no matter if the word is used metaphorically, or not. That is the reason why power is a central topic of Critical Discourse Analysis (CDA). CDA is an approach which uncovers *power* dynamics. Its main goal is to analyze how power is enacted, reproduced, and resisted, through text or speech (Van Dijk, 2001, p. 43). Critical discourse studies are specialized by their constitutive problem-oriented, interdisciplinary approach (Wodak and Meyer, 2015, p. 2) in research which not only describes the linguistic facts, but also contextualizes and problematizes them.

CMA (Cognitive Metaphor Analysis) is based on already mentioned Lakoff and Johnson's Conceptual Metaphor Theory and as Schröder (2021, p. 485) recalls, “shortly after its first success and diffusion, CMT has been adopted for critical discourse analysis”. Therefore CMT may be seen as a “fusion” of metaphor studies, cognitive linguistics, and critical discourse studies (Dirven et al., 2007; Schröder, 2021, etc.). As is well-known, CMT states that conceptual metaphors shape our thinking because of the mappings of the information between “source” and “target” domains in conceptual structure (Lakoff and Johnson, 1980/2003, p. 246). They shouldn't be underestimated and have to be critically analyzed because of their capacity to shape our thinking as a consequence of the existing interaction between the thoughts from the two domains (Charteris-Black, 2004, p. 27). The ability of metaphors to influence is mentioned by Mon et al. (2021) as they are reported as more engaging than their literal paraphrases. Metaphors are used deliberately in speech—“speakers use metaphor to persuade by combining the cognitive and linguistic resources at their disposal” (Charteris-Black, 2004, p. 11), and beyond the fact that they are a means of persuasion, they are inaccurate and misleading as “a metaphorical concept can keep us from focusing on other aspects of the concept that are inconsistent with that metaphor” (Lakoff and Johnson, 1980/2003, p. 10). In our contribution, we also take into consideration some theoretical observations made by scholars in the field of Rhetoric and Manipulation (Maillat and Oswald, 2009) and in the analysis of political discourse (Ilie, 2016; Mavrodieva, 2020).

## 3. Data

The data we use is collected and excerpted from media texts and political speeches as we are interested in public discourse and the representation of military language in the official communication during the COVID-19 crisis.

For which regards Italy, the analysis runs along three lines: reports of the titles from the main newspapers and from national radio and TV announcements; President Sergio Mattarella's speeches; and the opposition to the military rhetoric by some Italian associations and NGOs. On a descending grade, these three lines represent the different levels of the modulation of the military rhetoric in Italy, from the fullest embrace of the war-like rhetoric, to the Presidential speeches that directed the military discourse toward the necessity of a renewed unity and solidarity, to the clear stance of peace associations and NGOs that, from the very start of the pandemic, denounced the substantial and symbolical risks of the dissemination of such rhetoric. The first level is only reported here since there are many studies that, from different methodological and theoretical approaches, have collected a huge corpus of data on the war-like rhetoric in Italy (see e.g., Busso and Tordini, 2021; Elia, 2022), while the second and the third level are analyzed in detail to offer a more nuanced picture of the different ways in which the military rhetoric was mobilized—or opposed—in Italy.

Bulgarian corpus is taken from the biggest private national TV channels—bTV and NOVA as well as from another private TV channel—Bulgaria on air. Some collected written texts are published on the site of the Bulgarian National Radio, others are excerpted from popular news sites like DW, Focus, etc. They contain the Prime Minister's speeches, speeches from some other authorities, journalist materials about politicians' words and deeds, and some news about the spread of the pandemic in Bulgaria and abroad.

The Ukrainian official discourse on the problems of combating COVID-19 is analyzed on materials of public speeches presented on the official website “President of Ukraine Volodymyr Zelensky. Official online representation,” as well as interviews of the President with leading Ukrainian and foreign media during 2020–2021. For analysis, we also include publications of Ukrainian popular news sites, radio and and TV channels, posts by representatives of the Ukrainian authorities on the Facebook social network.

## 4. Analysis

### 4.1. Italy (Paola Giorgis)

In late February 2020, Italy was the first European country to be struck by the COVID-19 pandemic. We had heard some rumors in early/mid-February about a new virus that was circulating in China, but the general mood was that it was China, it was far away, and it would not affect us. Then, suddenly and ferociously, literally from 1 day to another, we were in.

COVID-19 was soon spreading fast in Italy—particularly in the North, with an appalling number of deaths. Strict measures were immediately taken, but they seemed of no use. Nobody knew what it should be done. There were no known procedures, and no protective devices—face masks, sanitizing gel, etc. To add to the overall chaos and uncertainty there came the titles of the headlines: “We are at war” [*Siamo in guerra*], “We are fighting at the forefront” [*Stiamo combattendo in prima linea*], “The enemy has invaded a defenseless country” [*Il nemico ha invaso un paese indifeso*]. Hospitals were trenches, the daily count of deaths and infected appeared every night in prime time as a war bulletin, doctors and nurses were celebrated as the “new heroes.” (Vovou, 2021).

A “pandemic” is defined by the World's Health Organization as “the worldwide spread of a new disease.” On the Oxford University Dictionary, “war” is defined as “an armed conflict between two different countries or different groups within the same country.” So, why was a disease narrated as an armed conflict?

There are some cognitive elements and socio-economic effects that can be pertinent both to a pandemic and a war, as I widely discuss in the entry “war” of the online dictionary *In Other Words* (Giorgis, 2020–2021). One of the most prominent connections is related to the randomness and the number of deaths. In Italy, the shocking evidence was the photograph shot at nighttime on March 18^th^, 2020. The photo showed a long column of military lorries carrying dozens of coffins from Bergamo to other cities in Italy: the number of deaths had been so high that the funeral homes could no longer deal with the burials. On the other hand, one of the most relevant structural differences between a war and a pandemic is that war is always the result of a deliberate political decision, while getting sick is not a matter of choice—here resounds Susan Sontag's critique on the use of war-like metaphors to define an illness (since, to begin with, “illness is *not* a metaphor”−1979, p. 3—and ultimately makes the sick victim both of the illness *and* the metaphor).

As in most countries, the use of military language in public and political discourse was pervasive in Italy, with a notable exception. The majority of President Sergio Mattarella's speeches did not mention war but, alluding to other difficult periods lived by the Italians in their history, sustained that precisely in those hard times the Italians showed their best qualities, building up a long story of solidarity and the creation of a community through and beyond the different crisis. All the speeches then retrace and appeal to those qualities, such as community-building, a spirit of unity, renovated solidarity and hope, resilience, and reconstruction toward a new beginning (e.g., March, 27^th^, 2020; June, 2^nd^ 2020; May 1^st^, 2020; April, 25^th^, 2021; May, 1^st^ 2021; June, 2^nd^ 2021)^3^.

The presidential speech that directly mentioned war—and COVID-19 as the enemy—was that of June 1^st^, 2020^4^. President Mattarella said that June 2^nd^ 1946 had marked the birth of the Republic as a new beginning after the divisions, the sufferings, and the destruction of war, tracing the path for a common destiny of democracy. Sustaining that the Italians have the quality and the strength to rebuild the country as they had done 70 years before, he was certain that the same communal spirit would pave the way to the rebirth of the nation after the pandemic.^5^

While Mattarella's speeches contained references to social and mutual responsibility and care, the military rhetoric continued to characterize the public and political discourse (as quoted above, see e.g., Busso and Tordini, 2021; Elia, 2022) to reach its momentum on March 1^st^, 2021, when the new Italian Prime Minister, Mario Draghi, appointed a general, Francesco Paolo Figliuolo, as Extraordinary COVID-19 Emergency Commissioner with the special task of managing the vaccine logistics. The general, who appeared on the public scene with his uniform vastly decorated with medals and insignia, thus became the visual embodiment of the war metaphor connected to the pandemic: to fight a war, it takes a general.

Since the early insurgence of the pandemic several anti-war movements, associations, NGOs, journalists, citizens, intellectuals^6^ publicly demanded with petitions and articles to stop using the military vocabulary to define the COVID-19 pandemic. Critical analysis and concerns sustained that not only militarizing language meant militarizing society, but also diverging the attention to what was mostly needed to contrast the pandemic—the implementation of efficient and local health and care systems. Anti-war movements, such as The Italian Network of Peace and Disarmament [*La rete italiana di pace e disarmo* (Rete Italiana Pace e Disarmo, n.d.)] and the Italian NGO Emergency (Emergency, n.d.) denounced that instead of investing more in health care, during the pandemic there had been an increase in the military expenses and investments.

Notwithstanding all debates, counterarguments, articles and petitions, the war metaphor remained a constant in Italian political and public discourse throughout 2020 and 2021. In April 2021, a further piece of war-like rhetoric was added to the public discourse: the war against the virus will be won only if the war of the vaccines is won. And to denounce the shortage of the vaccine supplying, vaccines were defined as “munitions” in several media—“we are running out of munitions” [“*stiamo esaurendo le munizioni*”], was the general cry. Underneath the surface of this metaphor, we can see the vaccine as a bullet inside our body, which becomes the battlefield of an invisible fight against an invisible enemy. Here, we can hear again Sontag's resolute warning against the use of war metaphors in health discourse.

Metaphors construe the meanings we give to experiences. In the final sentence of her essay *Illness as Metaphor*, Sontag (1979) sustains that “imposed” metaphors reveal our incapability to deal with the structural problems of our societies as well as with our fears and frailties, while it is our responsibility to be aware of the substantial moral and ethical weight of the words and metaphors that we use. A warning that, I would add, is particularly relevant at times of crisis.

### 4.2. Bulgaria (Bilyana Todorova)

The war-connected language use in Bulgaria started at the very beginning of the COVID-19 pandemic (March 2020–May 2020), but it was not necessarily metaphorical as there were government actions that were used as if there is a military threat.

The situation during these first days and months has been described in detail by BBB (Todorova, 2021a,b). In short, at the very beginning, before even the first COVID-19 case was confirmed, on February 26^th^, 2020 the National Operational Headquarters was announced (the date of the first confirmed cases was March 08, 2020). The word for “headquarters” in Bulgarian is “щаб.” It comes from German and its literal meanings are “Management of a military unit; A building housing such management” (RBE, 2021). The third, additional meaning is broader and may be translated as “A governing body of a party, organization, etc.” Moreover, the members of the National Operational Headquarters—the structure, mentioned above—were two military doctors, between them the Chair Prof. Mutafchiyski (who became publicly popular as “The General”). In most of the public appearances of “The General,” he wore his military uniform. Prof. Mutafchiyski became the “symbol” of the measures and he was the “face” of the COVID-19 government strategy as he gave briefings in his uniform every morning until May 2^nd^, 2020.

The militarization scenario was not presented only by the military uniforms and the morning briefings. Like many other authorities, the Bulgarian government enacted several measures and on March 13^th^, 2020 declared a “state of emergency” (*извънредно положение*). The measures were seen as controversial as people were forbidden to leave the district centers without special permission and there were checkpoints at the exits, they were not allowed to walk in parks, benches were dismantled to prevent people gatherings, and police cars were going around checking if there were rules violations.

The term “state of emergency” [*извънредно положение*] itself “is highly unclear although it is mentioned in the Bulgarian Constitution where the expression “military or another state of emergency” [*военно или друго извънредно положение*] was used without a clear definition of what it exactly means (Todorova, 2021a, p. 102). The opaqueness of the regulations and the suggestion that the Government actions are stricter than needed results in distrust of the Prime Minister as well as in a long-term skepticism of the seriousness of the COVID-19 pandemic.

The similarities between the presentation of the war-like situation and the COVID-19 crisis are not only in the above mentioned examples. As in many other countries, the Bulgarian politicians used war-like rhetoric in their speeches.

Prime Minister Boyko Borissov states during the Parliament debates as follows: “This is a bacteriological war^7^.” [*Това е бактериологична война*] (Debates, 2020) Some days later, on 17.03.2020 in the unplanned briefing, he also used a war metaphor: “We are in a war with an invisible enemy” [*Ние сме във война с невидим враг*] (Briefing, 2020). He is not the only one who preferred such a language: Ivan Geshev, the Prosecutor General, announced: “We should go[…]into a state of almost martial law”[*Трябва да се мине* *[…]* *в режим на почти военно положение*] (Ivan, 2020).

A study by Osenova (2021), who inspected a corpus of Parliamentary speeches for metaphoric uses connected to COVID-19 for the pandemic period (Nov. 2019–July 2020), reveals that the most frequent metaphor frames in the data are these of CONTROL (*recovery from COVID, dealing with COVID, overcoming / limiting COVID, controlling the epidemic, measures against the pandemic, prevention of the pandemic*) and WAR (*fight against COVID, protect citizens from COVID, summer will destroy the pandemic, the first line in the fight against the pandemic*, etc.).

What is important to be mentioned is the fact that some sports metaphors, control metaphors, and war metaphors are interrelated because they share not only the same vocabulary but they also represent a similar ideology: the relations of authority, power, distance, and pressure.

The war rhetoric includes the image of the Enemy. However, the Enemy is not only the virus, although this metaphor persists in the language of medical authorities, journalists, and politicians during all periods of the COVID-19 pandemic. At the same time, the image of another enemy has been consistently mentioned since the beginning of the crisis: irresponsible people. At the beginning of 2020, Prime Minister Borissov said that strong measures were necessary because of the “undisciplined people who spread the infection”. Later, after the beginning of the vaccination programme, because of the unwillingness of many people to vaccinate because of the mixed messages coming *via* social media and the so-called hybrid war, the enemy label is often put into them. For example, Dr Spiridonova said on November 18^th^ 2021: “We should limit our tolerance to unvaccinated people… Everything that happens to us is a test how we are prepared as society for extreme measures in conditions of war, because we are in a biological war” [“Трябва да ограничим толерирането на неваксинираните хора…Всичко, което ни се случва, е тест за обществото ни как сме подготвени и на екстремни мерки в условията на война, защото ние сме в биологична война.” (D-r Spiridonova, 2021)].

In 2021 there was a decrease in the use of military metaphors by politicians as the political situation was unstable and Bulgarians had to vote several times—in Parliamentary elections on April 4^th^, on July 11^th^, and on November 14^th^, in elections for a President on November 14^th^ and November 21st. Moreover, there were also partial local elections in some district towns. The insecurity and the lack of trust in politics as a whole made politicians more conscious of their language, so they avoided blaming ordinary citizens. However, according to Worldmeter, due to the reluctant measures of the caretaker governments, at the end of 2021 Bulgaria has the second-highest COVID-19 mortality rate in the world (Bulgaria has the Second, 2021).

War metaphor researchers agree that war metaphors are very useful for a short term mobilization of people, as they are a tool for increasing the consciousness about the importance of taking measures (Flusberg et al., 2017; Semino, 2021, etc.). However, when the measures are disproportionate and the danger seems not so imminently frightening, these metaphors and excessively strict restrictions lead to skepticism, distrust in the actions of the authorities, and a refusal to comply with any restrictions.

What about media texts? In the beginning, a large number of metaphoric uses have been found, for example:“Coronavirus death toll continues to rise” [*Продължава да расте броят на жертвите на коронавируса*] (bTV, Feb. 09, 2020); “Bulgaria is at war with COVID-19” [*България е във война с Ковид*-19**] (DW, Nov. 13, 2020), etc. Later some of them remain popular, for example, “he/she lose the battle with COVID-19,” but they become less frequent as a whole.

The situation in other countries is seen as very important to journalists and the articles, which are concerned with the measures abroad, have been regularly published, as follows: Slovenia defeated the coronavirus (May 15, 2020) [*Словения победи*^8^ *коронавируса*] (Slovenia, 2020), How Taiwan beat the coronavirus (Nov. 11, 2020) [*Как Тайван победи коронавируса*] (Kak Taivan, 2020), Coronavirus: how Portugal defeated the British variant (April 02, 2021) [*Коронавирус*:* как Португалия победи британския щам*] (Koronavirus, 2021), Denmark defeated COVID-19, they remove all restrictions. Sweden will repeal its measures at the end of September (Sept. 10, 2021) [*Дания победи Ковид*-19*, премахват всички ограничения. Швеция ще отмени мерките си в края на септември* (Daniya, 2021)], Iceland defeated COVID-19, it plans to return to normal life (Oct. 19, 2021) [*Исландия победи Ковид*-19*, планира връщане към нормалния живот*] (Islandiya, 2021), etc. However, all of these metaphorical titles have been seen as problematic as the pandemic, in fact, has not been overcome anywhere, although there were some countries which has governed the crisis better.

In the second part of 2021, war metaphors have been used when talking about measures and restrictions. As noted above, Bulgaria is the European country with the fewest vaccinated people, because of the popularity of disinformation and conspirative theories, and many people and some branches do not support any measures. For example, Richard Alibegov, the President of the Chamber of Restaurateurs, used the war metaphor in September when some measures are planned because of the increase of the COVID-19 positive tests numbers: “We are boycotting the order… If they want a war, they will have one.“ (Sept 02, 2021) [*Бойкотираме заповедта…Щом искат война, ще я имат* (Alibegov, 2021)].

Some organizations who don't believe that the COVID-19 crisis is a real pandemic find the measures dangerous, and they also use war metaphors, The use of military rhetoric by anti-vaccine activists in different countries, including Bulgaria, is discussed in some media texts (Antivaksarite se radikalizirat: Nyama koronavirus, 2021), for example, “This is a chemical war against our children (i.e., the use of disinfectants at schools) [*Това е химическа война срещу нашите деца*, Sept 19, 2021)], etc.

Conspiracy theories that deny measures against the virus, that present it as a “just virus,” or as a deliberately created in a laboratory to limit the rights and freedoms of citizens, that deny the effectiveness of vaccines and even proclaim their outright harmfulness, find a particularly good reception on social networks, where they have been spread uncritically. There are suspicions that the so-called troll factories (Mavrodieva, 2022), which began to publish anti-Ukrainian and pro-Russian content en masse after the outbreak of the war in Ukraine, are also to blame for their spread.

To sum up, the war rhetoric in Bulgaria is common when the topic is COVID-19. What is important is the fact that the boundary between literal and metaphorical uses is not always clear. Moreover, the war rhetoric at the beginning of the spread of the virus is one of the reasons for the subsequent skepticism. Mistrust in the motivation of institutions to take action has led to many casualties and to extremely low vaccination rates. As the crisis is a long journey, not a short battle, the war metaphor's popularity decreases over time and the frame used by authorities changes. More interestingly, military metaphors continue to be used by the opponents of the measures.

### 4.3. Ukraine (Olena Semenets)

Since 2014, Ukraine had been forced to restrain the military aggression of the Russian Federation in the east of the country. Therefore, during 2020–2021, Ukraine was experiencing two protracted crises at the same time: the long-drawn-out military conflict in the Donetsk and Luhansk regions and the COVID-19 pandemic.

In 2019, the new President Volodymyr Zelensky was elected in Ukraine. The rhetoric of his public speeches at first was largely based on show business technologies, in particular the techniques of humorous and satirical discourses (as Volodymyr Zelensky had considerable previous professional experience in the entertainment industry).

After the end of the first wave of the epidemic, at a press conference dedicated to the results of the first year of his presidential term (May 20^th^, 2020), Volodymyr Zelensky expressed confidence that the country had coped with a serious crisis related to COVID-19. He praised the work in this area, his own and of the Prime Minister Denis Shmygal: “... we are masters of sports in the fight against coronavirus. I'm sure of it. Take the statistics” [… *ми майстри спорту по боротьбі з коронавірусом. Я в цьому впевнений. Візьміть статистику*] (Pres-konferentsiia, 2020).

However, such “sports” rhetoric contrasted too much with the seriousness of the epidemic situation in Ukraine. This statement of the President was considered as a sign of an inexperienced politician's overconfidence and was severely criticized precisely on the basis of statistical indicators, to which Volodymyr Zelensky himself appealed (My—maistry sportu, 2020; Khozhainova, 2021; Komarova, 2021).

Later on, President Zelensky's anti-epidemic discourse became much more serious. This is manifested, in particular, in the use of “military” rhetoric in that discourse. Several stages of the development of such metaphorical rhetoric in the President's speeches can be distinguished.

August 2020. In his speech on the occasion of the Independence Day of Ukraine, Volodymyr Zelensky builds on a “military” metaphor, drawing a parallel between the spheres of reality: war—pandemic—economic crisis: “We are building just such a country! A country that is always ready to fight back. And it doesn't matter who attacks: the aggressor, the virus, the global crisis” [**M*и будуємо саме таку країну*!* Країну, яка завжди готова дати відсіч. І байдуже, хто атакує*:* агресор, вірус, світова криза*] (Promova Prezydenta, 2020).

November 2020. The metaphor deepens, covering specific areas of social and professional relations in Ukrainian society. In the greeting on the occasion of the Day of the social worker: “The coronavirus pandemic has significantly changed our lives. At the forefront of COVID-19's social consequences, social workers are at significant risk” [*Пандемія коронавірусу суттєво змінила наше життя. Перебуваючи на передовій боротьби із соціальними наслідками *COVID-19*, працівники соціальної сфери піддаються значній небезпеці*] (Vitannia Prezydenta, 2020).

December 2020. In the President's interview for the publication in Focus, the metaphor develops and branches out. Starting from the direct statement according to the model “*S is P”*: “Coronavirus is war” [*Коронавірус—це війна*]—to the defining in the subsequent story the directions of hard work as a struggle. The President explains the change of the three health ministers by “the psychological killing force” of the virus: “I consider that the virus killed the ministers psychologically. They couldn't do the job very quickly, not because they were bad, but because they were ministers at the time” [*Я вважаю, що вірус психологічно вбивав міністрів. Вони не могли дуже швидко виконувати завдання не тому, що погані, а тому що були міністрами в такий час*] (Shashkova, 2020). Then, in the full interview, the President's discourse of the struggle for a vaccine further develops.

The metaphor of war was completely legitimate, first of all, in the discourse of the physicians themselves, in their professional assessment of the situation: “… we are here just like at war. Doctors, nurses, paramedics—all work for the good to help people” [… *ми тут просто як на війні. Лікарі, медсестри, санітарочки—всі працюють на благо, щоб допомогти людям*], March 2021 (Sadovyi, 2021); “You have to gather strength even in spite of tears: you came out crying—and you go to the sick again. We now have two frontlines—at the battle line and in medicine” [*Доводиться набиратися сил навіть через сльози*:* вийшов поплакав—і знову йдеш до хворих. У нас зараз дві передові—на фронті й у медицині*], September 2021 (Chyrytsia, 2021).

In general, the metaphor “war against the coronavirus” has not become as widespread in Ukrainian official, political, and media discursive practices during 2020–2021 as in other Western countries (Semenets, 2022). The word “war” in the public and personal discourses of Ukrainians was used primarily not in the metaphorical, but in the direct, denotative sense: “war” as “the armed conflict in eastern Ukraine, the resistance to external Russian aggression.”

An indicator of this state of public consciousness could be seen in the awarding of the national prize “Global Teacher Prize Ukraine” in 2021 which for the first time referred to the nomination category “Teacher Working in the Combat Zone.” The writer Serhiy Zhadan, who presented the award to a teacher from the combat zone, stressed: “Teachers of Donetsk and Luhansk regions hold an equally important line of defense” [*Вчителі Донеччини та Луганщини тримають не менш важливу лінію оборони*]. The winning teacher herself noted: “This is the first of such nominations. And my most cherished dream is for it to be the last. That we never had teachers working in the combat zone. And we were just teachers of Ukraine” [*Це перша така номінація*. A* моя найзаповітніша мрія, щоб вона була останньою. Щоб ніколи у нас не було вчителів прифронтової зони*. A* ми просто були вчителі України*] (Global Teacher Prize: naikrashchym stav vchytel ukrainskoi Artur Prodaikov, 2021).

Speaking at the debate of the 75^th^ session of the UN General Assembly on September 23^rd^, 2020, President of Ukraine Volodymyr Zelensky focused on the growing challenges to modern world security and the situation of war that Ukraine has been experiencing since 2014: “I speak of this as the Head of State in which the Russian Federation annexed the Crimean Peninsula in the 21^st^ century. A state that has been deterring its military aggression in Donbas for 7 years. How would the founders of the United Nations feel if they learned that 75 years later there would be a war in central Europe?” (Vystup Prezydenta, 2020).

The Russian aggression against Ukraine since 2014 has the character of a hybrid war. The armed confrontation is accompanied by hard Russian propaganda and constant information attacks. The concept of “war” in the minds of modern Ukrainians is primarily associated with countering Russian aggression in eastern Ukraine and information warfare.

One of the important aspects of the information struggle in the Ukrainian media environment in 2020–2021 was represented by the metaphorical field of “battle of vaccines” and “battle for the vaccine”.

The metaphor “battle of vaccines” [“*битва вакцин*”] means “tough competition, fierce struggle between vaccines.” The word “vaccine” in this phrase means not the drug itself, but—based on metonymic connection and personification, i.e., on the basis of metonymic metaphor—it means those collective subjects that own or dispose of this drug: pharmaceutical companies, certain countries, authorities in those countries.

In the metaphor “battle for the vaccine” [“*битва за вакцину*”] the dependent noun has the meaning of the object being fought for. During 2020, that metaphorical phrase was used mainly in the meanings:

- Invention, testing, and production of vaccines.
- Purchase, receiving the vaccine.

However, in January 2021, in Ukrainian media discourses, the semantic volume of the metaphor was supplemented with new components. Characteristics of the quality of information in media space had also become the constituents of the “battle for the vaccine”. At this time, there were demands that the government's actions to purchase vaccines must be transparent as well as claims that a high-quality information campaign on the need for vaccination and the promulgation of a clear vaccination mechanism throughout the country were needed. All of this together was also part of the “battle for the vaccine” (Semenets, 2022). Another important meaning of the phrase “battle for the vaccine” was the fight for the fair distribution of vaccines between countries, including free access to the global initiative COVAX (Skandal u Yevropi, 2021).

The information environment of discussions on vaccination during the pandemic was a battleground for geopolitical, economic, informational influence, i.e., the sphere of information warfare. The discrediting of Pfizer, Moderna, AstraZeneca vaccines and, in contrast, the positive coverage of the Russian “Sputnik V” vaccine was carried out primarily by Russian media and Ukrainian media with strong pro-Russian rhetoric, as well as pro-Russian deputies and popular bloggers.

The very name of the vaccine “Sputnik V” contains direct reference for the Cold War. The vaccine is named after the first orbital satellite launched by the Soviet Union in 1957 and started the global space race. Kirill Dmitriev, head of the Russian Direct Investment Fund, which is financing Russian vaccine research, suggested the name. Referring to the world's first spacecraft launched by the USSR, in late July 2020 he said to CNN: “Americans were surprised when they heard Sputnik's beeping. It's the same with this vaccine. Russia will have got there first” (Chance, 2020). The Russian authorities considered this vaccine as a powerful weapon of information warfare and saw a military content potential in it. In 2021, on the eve of Victory Day on May 9^th^, Vladimir Putin compared “Sputnik V” to Soviet-era weapons, arguing that the Russian vaccine was “as reliable as a Kalashnikov assault rifle” (Putin Porivniav, 2021).

In his interview with The New York Times on December 16^th^, 2020, the President of Ukraine Volodymyr Zelensky identified quality as the main criterion for choosing a vaccine for Ukrainians, emphasizing that the promotion of “Sputnik V” is “ones more strongest information war by Russia” [*ще одна найсильніша інформаційна війна з боку *P*осії*]. The issue of vaccine quality was key: “… we must not allow Ukraine to take the Russian vaccine that has not passed all the tests. We have no real evidence that that vaccine has a hundred-per-cent positive effect. … Ukraine primarily bases its decision on choosing a safe vaccine” (Interviu Volodymyra Zelenskoho, 2020).

Thus, “battle of vaccines” and “battle for the vaccine” as a metaphorical field of information struggle demonstrates fundamentally different approaches from the Russian Federation and Ukraine, highlighting different values and semantic dominants in official, political, and media discursive practices.

## 5. Discussion

Discourse analysis on the rhetoric of COVID-19 pandemic has produced such a wide range of studies that it has become a genre in itself. And being the “war metaphor” the most widely used across the world, it has occupied a large place of its own within such a genre, with comments ranging from the most neutral or moderate to the most critical, as we have presented in this contribution.

In the pages above, we have discussed how the war metaphor has been mobilized in political and media discourses in the three countries where we live—Italy, Bulgaria, and Ukraine. From a cross-cultural perspective, this complies to one of the possible research methods and culture sampling (van de Vijver, 2001, p. 3002). Cross-cultural studies “involve persons from different countries and/or ethnic groups; a defining characteristic is their comparative nature” (*ib.*, p. 2999). These studies sustain that “groups with a different cultural background tend to differ on a variety of outcome-relevant characteristcs” (*id*.).

From a cross-cultural perspective, in our contribution we can notice that though the military discourse propagated in our countries presents some common threads—an appeal to unity, the mobilitization against a common threat, the rhetoric construction (or reconstruction) of a specific national identity, the legitimization of security measures—the historical, cultural, and political context of each country framed and signified the war metaphor in different ways, following different strategies and enacting different argumentative functions.

In Italy, due to the historical past of the country marked by Fascism, the national and public rhetoric mobilized the war metaphor directing it toward what is culturally perceived as “the good war” that is the war of Liberation from Nazi and Fascist regimes, a sort of “national redemption” after the fall into Fascism. References to war thus mainly played on the sense of a renewed national solidarity and unity to be attained during and after the COVID-19 pandemic as it was attained during the war of Liberation and after the destructions and internal divisions created by Fascism. The military presence though became blatantly visible during the first, strictest, lockdown (March, 9^th^-May, 18^th^ 2020), when military forces in uniforms or camouflage patrolled the streets, and with the appointment of a general to manage the vaccine logistics (March 1^st^, 2021). While the first generated an overall great impression in a public opinion unaccustomed to see militaries in the streets, reviving bad memories in older generations and dismay in those born after World War II, the latter was received with rather opposite sentiments, with the institutions and the traditional media saluting and celebrating the military efficiency, while the social media displayed a wide array of ironic and sarcastic comments.

From the very start of the pandemic, in Bulgaria the use of the military rhetoric was less a rhetorical move and more a clear political stance—e.g., through the presence of military doctors on TV and the daily 8 am briefings televised by a general in uniform. Again, it is interesting to notice how the war metaphor was filled in by specific cultural and historical elements. A consolidated lack of trust in politics and politicians combined with distrust in authorities, created a rather peculiar occurrence. As the war rhetoric implies the construction of an enemy, in Bulgarian political discourse the enemy doubled, being not solely the virus itself, but also those who performed irresponsible behaviors: those who refused to comply with restrictions first, and to vaccinate then.

In Ukraine, where a military conflict had been a constant presence since 2014, at the beginning of COVID-19 pandemic two different phenomena could be observed. On the one hand, the use of the war metaphor was not so active since, to paraphrase Sontag, “war” was *not* a metaphor but a real armed conflict in eastern Ukraine. At the start of the pandemic, the Ukrainian president preferred to use sports metaphors to define COVID-19 in his discourse. On the other hand, when the war metaphor emerged in political speeches, the enemy tripled—the aggressor, the virus, and the economical crises. A further—and highly contextualized—step in the Ukrainian war rhetoric was the information warfare on the vaccines, which saw a sort of reproposal of the Cold War, here engaged between Western vaccines and the Russian Sputnik V, multiplied by the violence of Russia's informational aggression against Ukraine.

As we have seen, a relevant factor in the war metaphor is the construction of a specific enemy (or enemies), a major actor that is evoked and mobilized for specific political purposes that manipulate and bend to their own interest specific cultural and historical factors. Another element that lies at the core of the war metaphor, and actually nurtures its deepest roots, is the emotional appeal to fear that, as Wodak (2015) has discussed, is a major player in political rhetoric. In the discourses of the three countries taken into consideration, fear occupies a central role though, in this case, with some recurrent similarities, such as for example the progressive shift from the “fear of the virus” to the “fear of the vaccine” caused by a general scorn or mistrust in the authorities, or by the concern of being “invaded” by the enemy vaccine. Another main actor of the war metaphor is the figure of the “hero” who bravely fights at the frontline. While in most countries doctors and nurses were those saluted as the “new heroes,” it is interesting to notice that in Ukraine health professionals (as well as teachers working in the combat zones of the Donetsk and Luhansk regions) when using such a metaphor were compared not with legendary archetypal “heroes,” but with quite real Ukrainian soldiers who protected the country from Russian armed aggression in the east of Ukraine.

As we can notice from these considerations, though a most globally used metaphor, the “war metaphor” both evoked and responded to specific national issues, concerns, cultural and social situation, historical memories, ideologies, knowledge about the dominant forms of discourse in society (Kövecses, 2015, pp. 181–186). If “using metaphorical language is joint action that requires a common ground” (*ib*., p. 179), such common ground is highly influenced by diverse contextual factors, including that of extending and reapplying metaphors previously used. In the last decades, the neoliberal governments have applied the war metaphor to various domains—e.g., war on crime, war on drugs, war on AIDS, war on terrorism—creating a logic of perennial war that has justified measures of securitazion and control of the bodies, disseminating “a discourse on the normalization and naturalization of ongoing violence” (Dias and Deluchey, 2020, p. 3) where:

- War and peace become synonymous, as well as exception and rule, coup d'état and governance, politics,
- And police, neoliberalism and civil war. This is why, first and foremost, this war is communicational and,
- Involves the corrosion and misrepresentation of language, the perversion of enunciation and a systematic,
- Inversion of the value of the words and the meaning of discourse itself (*id*.).

## 6. A final note

In late February-early March 2022, while we were finalizing this contribution on the war metaphor, a *real* full scale war, caused by the invasion of Ukraine by Russian troops, broke out in the heart of Europe. As for many other contemporary wars and conflicts (Yemen, Somalia, Myanmar, Syria, Sudan, only to name a few), civilians are those who pay the highest price in terms of casualties, suffering, and displacements. Divisive narrations highly contribute to fueling the flames of hate and constructing the Enemy. As linguists, it is our responsibility to raise awareness on the mechanisms of such rhetorical strategies to deflame such narratives, exposing and deconstructing the textual and visual mechanisms that disseminate discriminatory language, but also imagining creative proposals to subvert polarized discourses.

## Data availability statement

The original contributions presented in the study are included in the article/supplementary material, further inquiries can be directed to the corresponding authors.

## Author contributions

This contribution, fully shared by the three authors, was drawn up as follows: 1. Introduction, 2. Methodology/theoretical background, 3. Data, and 5. Discussion were co-written by the three authors. 4.1. Italy was written by PG. 4.2. Bulgaria was written by BT. 4.3. Ukraine was written by OS. All authors contributed to the article and approved the submitted version.

## Footnotes

^1^https://dictionary.cambridge.org/dictionary/english/war. It is not only in English—such meanings are described in French Le Robert (https://dictionnaire.lerobert.com/definition/guerre); Bulgarian RBE https://tinyurl.com/59p6xdtr and many others.

^2^During the invasion of Ukraine by the Russian troops in late February 2022, there were three words that, at the risk of jail or worse, could not appear in the Russian media: “war,” “invasion,” “attack” (The Guardian, 2022). The expression to be used was ‘special military operation'. We can thus see that while the pandemic has been narrated as “war,” “invasion,” “attack,” a *real* war was narrated as an “special military operation” https://www.theguardian.com/news/audio/2022/mar/04/what-russians-are-being-told-about-the-war-in-ukraine (accessed March 4, 2022).

^3^Besides the dedicated speech on COVID-19 on March 27^th^, 2020, the other presidential speeches analyzed were chosen for their national-symbolical relevance as they were pronounced on three major national days: April 25^th^ marks the liberation from the Nazi-Fascist regime (1945), May 1^st^ is Labour's Day, and June 2^nd^ marks the birth of the Italian Republic (1946) (Presidenza Della Repubblica, 2015).

^4^Mattarella anticipated on June 1^st^ his presidential speech of June 2^nd^ since the day after he visited Codogno, the town in Lombardy where there had been the first Italian case of COVID-19, and that had paid a high tribute of deaths.

^5^In the same spirit was Queen Elizabeth's speech to the nation on April 5^th^, 2020 (https://www.youtube.com/watch?v=2klmuggOElE). She recalled her first speech to the nation in 1940 when many families were separated, while the use of the sentence ‘We'll meet again' pointed directly to the title of Vera Lynn's famous song of 1939 that, during World War Two, symbolized the resilience of the British. After the Queen's speech, the song enjoyed a renovated fame, was used for charity funding events for the NHS, and reached a high position in the UK charts.

^6^The list of reactions is too long to be reported here. For the Italian context, please refer to Milesi (2020) “The virality of the military language” [*La viraltà del linguaggio bellico*] published on the online magazine *Vita*, where linguists, journalists and writers discuss about the reasons and the dangers related to the use of the war metaphor to speak about a pandemic. In the UK, in April 2020, the University of Lancaster launched a collaborative initiative to ask linguist experts and anyone who wished to engage in proposing “examples of inspirational non-war-related metaphors” (https://www.lancaster.ac.uk/linguistics/news/beyond-the-battle-far-from-the-frontline-a-call-for-alternative-ways-of-talking-about-covid-19). Such a joint endeavor then resulted in a comprehensive publication (Olza et al., 2021) that discusses critically the pervasiveness of the war metaphor in different countries. Another collective initiative is that of #ReframeCovid that was launched on Twitter among linguists and citizens from all over the world to promote non-war-related language on COVID-19.

^7^Boyko Borissov made a mistake: COVID-19 is a virus, not a bacterial infection. However, his words are cited here as an example of the representation of the pandemics as a war.

^8^In all these cases the verb ‘*победя*' is used—it means ‘win' and it is used primarily in military context, and later in sport and in everyday situations, including in medicine.
